# Temporal and habitat-specific variations in drivers of aboveground biomass dynamics in a Chinese subtropical forest

**DOI:** 10.3389/fpls.2024.1531654

**Published:** 2025-01-21

**Authors:** Yuxuan Bian, Qi Wu, Rong Zheng, Jiaqin Fu, Jianhua Chen, Xiangcheng Mi, Mingjian Yu, Yunquan Wang

**Affiliations:** ^1^ College of Life Sciences, Zhejiang Normal University, Jinhua, China; ^2^ Zhejiang Qianjiangyuan Forest Biodiversity National Observation and Research Station, State Key Laboratory of Vegetation and Environmental Change, Institute of Botany, The Chinese Academy of Sciences, Beijing, China; ^3^ College of Life Sciences, Zhejiang University, Hangzhou, China; ^4^ The Administration Center of Zhejiang Jiulongshan National Nature Reserve, Lishui, China

**Keywords:** ecosystem functioning, functional diversity, evolutionary diversity, structural diversity, niche complementarity, disturbance

## Abstract

Understanding the mechanisms governing biodiversity-biomass relationships across temporal and spatial scales is essential for elucidating how abiotic and biotic factors influence ecosystem function in natural forests. However, the simultaneous contributions of multiple abiotic (e.g., topography) and biotic factors (e.g., structural diversity) to aboveground biomass dynamics (ΔAGB) over time and across habitat types remain inadequately understood. To address this gap, we evaluated changes in aboveground biomass across a decade and various habitats, disentangling the relative influences of topography and multidimensional diversity on ΔAGB through datasets from forest inventories conducted between 2007 and 2017, along with phylogenetic relatedness, functional traits, and environmental variables from a subtropical forest in China. Our findings indicate that aboveground biomass at community level experienced a significant decline followed by an increase over the decade, predominantly driven by changes in the low-valley habitat. In contrast, no statistically significant alterations were detected in the aboveground biomass of mid-hillside and high-ridge habitats. Furthermore, the determinants of ΔAGB exhibited temporal variation. During the 2007-2012 period, ΔAGB was primarily influenced by functional and structural diversity, accounting for 66.11% and 21.35% of relative importance, respectively. In the subsequent 2012-2017 period, phylogenetic and structural diversity emerged as key factors, explaining 48.46% and 36.43% of relative importance, respectively. Additionally, we observed that the drivers and effects impacting ΔAGB exhibited significant variability across different habitat types. In summary, our study underscores the significant spatiotemporal dependence of abiotic and biotic drivers on biomass dynamics within forest ecosystems, thereby enhancing our understanding of the complex biodiversity-ecosystem functioning relationships.

## 1 Introduction

Anthropogenic global climate change and habitat destruction have exacerbated biodiversity loss worldwide (Synes et al., 2020; Richardson et al., 2023), resulting in irreversible negative impacts on species coexistence, services and functions of ecosystems (Isbell et al., 2017; He et al., 2024). Given that forests are indispensable to the worldwide carbon cycle and maintenance of carbon neutrality (Canadell and Raupach, 2008; Pan et al., 2011), the biodiversity-ecosystem functioning (BEF) relationships in forest communities have garnered considerable attention and pose a significant challenge in ecology (Gamfeldt et al., 2013; Liang et al., 2016; Ray et al., 2023; Zemp et al., 2023). Theoretically, a positive BEF relationship is expected when biodiversity promotes niche complementarity (i.e. the complementary effect) or the average competitive ability of species (i.e. the positive selection effect) (Lasky et al., 2014). Alternatively, a negative BEF relationship may occur if increased biodiversity results in a decrease of the average competitive ability of species (i.e. the negative selection effect) (Huston, 1997; Tilman, 1999). However, the relationship between biodiversity and aboveground biomass (AGB) within forest communities is complex and context-dependent, and potentially varying over time and across spatial scales (Cardinale et al., 2007; Reich et al., 2012; Forrester and Bauhus, 2016; Gottschall et al., 2022). It remains ambiguous how multiple abiotic and biotic factors simultaneously contribute to aboveground biomass dynamics (ΔAGB) over temporal scales and across various habitat types.

Considering multiple dimensions of biodiversity concurrently is able to facilitate a comprehensive and accurate insight into the ecological mechanism underlying BEF relationships (Cadotte et al., 2009; Srivastava et al., 2012; Wang et al., 2024). Previous researchers have already investigated how species and functional diversity influence AGB, revealing that species richness alone may inadequately capture the ecological differences or similarities among species (van der Sande et al., 2017; Yuan et al., 2019). In comparison to species diversity, functional diversity plays a more crucial role in ecosystem functions by reflecting a suite of core attributes essential for plant growth and reproduction within a community (Petchey and Gaston, 2002; Lian et al., 2022). A higher diversity of traits related to resource uptake enables a community to utilize resources more effectively, with resource use complementarity serving as an underlying mechanism linking functional diversity to ecosystem function (Fotis et al., 2018; Chen et al., 2023).

Structural diversity and phylogenetic diversity have been recognized as critical drivers of ecosystem functions (Dănescu et al., 2016; Yuan et al., 2018; Yang et al., 2024), as they offer insights into resource use efficiency and evolutionary history in forest communities, respectively (Cadotte et al., 2009; Zhu et al., 2021). Notably, due to its capacity to estimate both the actual volumetric occupancy and arrangement within niche spaces, stand structure is increasingly acknowledged in the context of the BEF relationship (Ali, 2019; Chen et al., 2023). Although it has been demonstrated that changes in BEF relationships may arise from the complementary trends in resource use strategies among species over time (Huang et al., 2018; Zheng et al., 2024), our understanding of how multiple dimensions of biodiversity contribute to ΔAGB over time remains limited. In the context of climate change, exploring the impact of multidimensional biodiversity on ΔAGB across temporal scales in natural forests is essential for elucidating the variation in biodiversity effects (Kardol et al., 2018).

The critical role of abiotic factors in shaping BEF relationships has been extensively examined (Ferry et al., 2010; McEwan et al., 2011; Quesada et al., 2012; Werner and Homeier, 2015). The “multivariate productivity-diversity hypothesis” posits that environmental conditions indirectly influence community productivity by affecting species diversity, thereby providing a theoretical framework for understanding spatial variation in BEF relationships (Cardinale et al., 2009). Habitat types comprehensively reflect topographic factors (e.g., elevation, slope, aspect, and convexity), which mediate microclimates and soil nutrients (Man et al., 2011; Lin et al., 2012), thus potentially impacting the ΔAGB of forests both directly or indirectly (McEwan et al., 2011; Lin et al., 2012; Zhang et al., 2024). For instance, certain habitat types such as ridges and steep slopes may experience periodic water stress, poor soil nutrients availability, and strong winds, where only species with stress-tolerant life history strategies can thrive (Paoli, 2006; Tanner et al., 2014). Distinct environmental factors across various habitats influence the species composition of plant communities as well as the growth performance of species, which in turn indirectly affect the AGB within forest communities (Chadwick and Asner, 2016; Jucker et al., 2018). Therefore, we argue that considering habitat types could further elucidate the spatial variation in diversity-ΔAGB relationships.

Subtropical forests, despite their relatively limited global distribution (Fang et al., 2001), rank second only to tropical forests in terms of species richness and serve as a significant carbon sink on the earth (Houghton, 2005; Piao et al., 2009; Li et al., 2019). They are essential in the worldwide carbon cycle and climate regulation (Yu et al., 2014). The forest within the Gutianshan National Nature Reserve exemplifies typical subtropical evergreen broad-leaved mature forest of China (Jiang et al., 2022), and large sustained forest dynamics monitoring plots with systematic vegetation inventories here facilitate the critical framework for linking abiotic and biotic drivers of carbon dynamics to spatiotemporal variation (Lin et al., 2012; Mi et al., 2021). To evaluate the potential contributions of multiple abiotic and biotic factors to ΔAGB over time and across habitat types in the subtropical forest, we examined the changes of AGB over time and across habitat types, disentangling the comparative impacts of these factors on ΔAGB across different temporal periods and habitat types. Specifically, we focused on the following three questions: (1) How did the AGB vary over time and across habitats during the past decade? (2) How did the abiotic and biotic determinants and their influences on ΔAGB differ across temporal periods? and (3) how did they vary across different habitat types?

## 2 Materials and methods

### 2.1 Study site and plot data

Our study was conducted within the Gutianshan National Nature Reserve in Quzhou City, Zhejiang Province, southeastern China, with a total area of 8107 hectares. The reserve is distinguished by its subtropical humid monsoon climate, exhibiting an average annual temperature of 15.3 °C and an average annual precipitation of 1963.7 mm (Yu et al., 2001). The predominant soil types in the area comprise red soil, yellow-red soil, red-yellow soil, as well as swamp soil, with a pH ranging mostly between 5.5 and 6.5. The evergreen broad-leaved forest, dominated by *Castanopsis eyrei* and *Schima superba*, is the main vegetation type in Gutianshan, commonly found below 800 meters and characterized as typical subtropical zonal vegetation (Yu et al., 2001; Legendre et al., 2009).

The 5-ha forest plot was established in 2002 according to the standard of the CTFS-ForestGEO protocol. The plot spans 200 meters in an east-west direction and 250 meters in a north-south orientation, containing two hillsides on the northern and southern sides and a valley in the middle, with a cross-section resembling an irregular “V” shape (Jiang et al., 2022). In this plot, all woody stems with DBH (diameter at breast height, 1.3 m) ≥ 1 cm were tagged, spatially mapped, identified to species, and measured DBH, number of sprouts and branches. Tree census is conducted every 5 years for the 5-ha long-term forest dynamics monitoring plot. More than 18000 free-standing individuals belonging to 161 plant species were recorded during the 2007-2017 period (**Table 1**).

**Table 1:** The species richness and individual abundance of the whole plot and different habitat types from 2007 to 2017.

Year	Whole plot		Low-valley habitat		Mid-hillside habitat		High-ridge habitat	
	total	mean ± sd	total	mean ± sd	total	mean ± sd	total	mean ± sd
Species richness								
2007	149	32.98 ± 7.31	133	31.90 ± 6.50	133	32.51 ± 8.21	98	36 ± 6.01
2012	159	34.66 ± 8.67	144	32.68 ± 6.96	140	34.6 ± 10.12	113	38.72 ± 7.19
2017	160	33.15 ± 8.53	143	30.76 ± 6.72	141	32.35 ± 8.70	115	39.36 ± 8.30
Individual abundance								
2007	17673	147.28 ± 49.49	6828	136.56 ± 44.45	6030	134 ± 45.25	4815	192.6 ± 37.71
2012	18901	157.51 ± 54.52	7295	145.9 ± 47.02	6443	143.18 ± 53.20	5163	206.52 ± 39.88
2017	16143	134.53 ± 51.05	5849	116.98 ± 37.18	5431	120.69 ± 43.42	4863	194.52 ± 41.06

### 2.2 Estimation of aboveground biomass

To estimate aboveground biomass (AGB), the tree height was first calculated by referring to Lin et al. (2012) (Equation 1). There were 47 species-specific tree height equations fitted in our study site, and an equation based on combined data from all species was used for the remaining 114 species. Then the AGB of each tree and branch was calculated by using the allometric growth equation improved by Chave et al. (2014) (Equation 2):


(1) $$\begin{array}{c} \;H=aD^{b}\times CF \end{array}$$

(2) $$\begin{array}{c} AGB\;=0.0673\times \;{\left(WD\times D^{2}\times H\right)}^{0.976} \end{array}$$

Where *D* is DBH (cm), *a* and *b* are estimated species-specific coefficients and CF is the correction factor. The wood density (WD) was obtained mainly from Liu (2012) *in-situ* measured data and through the search of the TRY database (Kattge et al., 2020). Among them, 14.9% of species without WD data were replaced by the mean WD of species of the same genus or family in the same climate region.

### 2.3 Abiotic variables

We defined abiotic factors within the context of topography and habitat types in our study. Four topographic variables were calculated (i.e. elevation, slope, aspect, and convexity) for every 20 m × 20 m plot following Harms et al. (2001). To test how the drivers of ΔAGB vary with habitat types, the 5-ha forest plot was divided into three habitat types at 20 m × 20 m scale (The extra 20 m × 10 m is not included): low-valley (H1, 50 plots), mid-hillside (H2, 45 plots), and high-ridge (H3, 25 plots). More detailed information about habitat classification could be found in Jiang et al. (2022).

### 2.4 Biotic variables

To test the impacts of biotic factors on ΔAGB, we measured four dimensions of biodiversity indices: species (taxonomic) diversity, structural diversity, phylogenetic diversity, and functional diversity. Species diversity was measured by Shannon-Wiener index (*H*), Simpson index (*D*) and Pielou evenness index (*J*) (Ma and Liu, 1994). The change values of Shannon-Wiener index (cH), Simpson index (cD), and Pielou evenness index (cJ) were represented as the differences between each 5-year period (the same below). Stand density (SD) and the coefficient of variation of DBH (CV_DBH_) were calculated for structural diversity variables (Ren et al., 2021). SD was the number of individual plants with DBH ≥1 cm per unit area (quadrat). The equation for the CV_DBH_ is as follows (Zhang and Chen, 2015):


(3) $$\begin{array}{c} CV_{DBH}=\sigma /\mu \end{array}$$

Where *σ* is the standard deviation of DBH in the quadrat, *μ* is the mean DBH in the quadrat, and the change values of SD (cSD) and CV_DBH_ (cCV_DBH_) were calculated at the same time.

According to the Angiosperm Phylogeny Group IV (APG IV), a phylogenetic tree was first constructed (Jin and Qian, 2022), and then the mean pairwise distance (MPD) and mean nearest taxon distance (MNTD) were calculated for all species in the community (Webb et al., 2002; Swenson et al., 2006):


(4) $$\begin{array}{c} MPD\;=\;\frac{MPD_{sample}-meanMPD_{null}}{sdMPD_{null}} \end{array}$$

(5) $$\begin{array}{c} MNTD\;=\;\frac{MNTD_{sample}-meanMNTD_{null}}{sdMNTD_{null}} \end{array}$$

Where *MPD_sample_* and *MNTD_sample_* are the actual observed values, while *MPD_null_* and *MNTD_null_* are the values of MPD and MNTD for randomly generated null communities under the null model, *sdMPD_null_* and *sdMNTD_null_* are the standard deviations of these values, *meanMPD_null_* and *meanMNTD_null_* are the average of these values. The change values of MPD (cMPD) and MNTD (cMNTD) were also calculated.

The WD, maximum tree height, and life form of plant species are closely related to forest AGB and are considered important functional traits (Ruiz-Benito et al., 2014; Ouyang et al., 2019). Therefore, we used these three types of functional traits to assess functional diversity. The WD used the data previously employed for calculating the AGB. The maximum tree height and species life form data were sourced from the Flora of Zhejiang (New Edition) (Li, 2021) and Flora of China (Wu et al., 1994-2009), with life forms categorized into three types: evergreen broad-leaved, deciduous broad-leaved, and coniferous. Then we calculated Rao’s quadratic entropy (RaoQ) and Functional dispersion (FDis) for functional diversity (Laliberté and Legendre, 2010). And the trait mean pairwise distance (traitMPD) and trait mean nearest taxon distance (traitMNTD) were calculated based on the functional trait dendrogram (Swenson et al., 2006; Shui et al., 2022):


(6) $$\begin{array}{c} traitMPD\;=\;-1\times \frac{traitMPD_{sample}-meantraitMPD_{null}}{sdtraitMPD_{null}} \end{array}$$

(7) $$\begin{array}{c} traitMNTD\;=\;-1\times \frac{traitMNTD_{sample}-meantraitMNTD_{null}}{sdtraitMNTD_{null}} \end{array}$$

Where *traitMPD_sample_* and *traitMNTD_sample_* are the actual observed values, while *traitMPD_null_* and *traitMNTD_null_* are the values of traitMPD and traitMNTD for randomly generated null communities under the null model, *sdtraitMPD_null_* and *sdtraitMNTD_null_* are the standard deviations of these values, *meantraitMPD_null_* and *meantraitMNTD_null_* are the average of these values. The change values of FDis (cFDis), RaoQ (cRaoQ), traitMPD (ctraitMPD) and traitMNTD (ctraitMNTD) were also calculated.

### 2.5 Statistical analysis

First, we used the Wilcoxon rank-sum test to determine if there were significant differences in AGB across the three tree censuses. Second, the generalized linear model was used to examine the impact of biotic (including the change values of them) and abiotic variables on ΔAGB. The ΔAGB was represented as the difference in AGB between each 5-year period. We divided the study decade (2007-2017) into two 5-year periods (2007-2012 and 2012-2017) to investigate the temporal changes in the drivers of ΔAGB. Furthermore, the plot was categorized into three habitats, and we examined the relationship between the explanatory variables and the response variables within them. The initial values and the change values were used for all biotic variables. To enhance the comparative analysis among drivers and models, all variables were scaled and variables with too high collinearity of variance inflation factor (VIF) >5 were removed (Fox and Weisberg, 2018). Finally, we selected our optimal models with lowest Akaike information criterion (AIC) (Bartoń, 2012), then performed hierarchical partition on them to assess the relative importance of all variables influencing ΔAGB (Lai et al., 2022; Lai et al., 2023). Some variables are not represented in our figures because they are not included in the optimal model. The initial data proofreading and organization were completed in Excel 16.0, while the subsequent calculations of indices, data analysis, and plotting were all conducted in R (version 4.2.2).

## 3 Results

### 3.1 The changes of aboveground biomass over time and habitats

**Figure 1:**
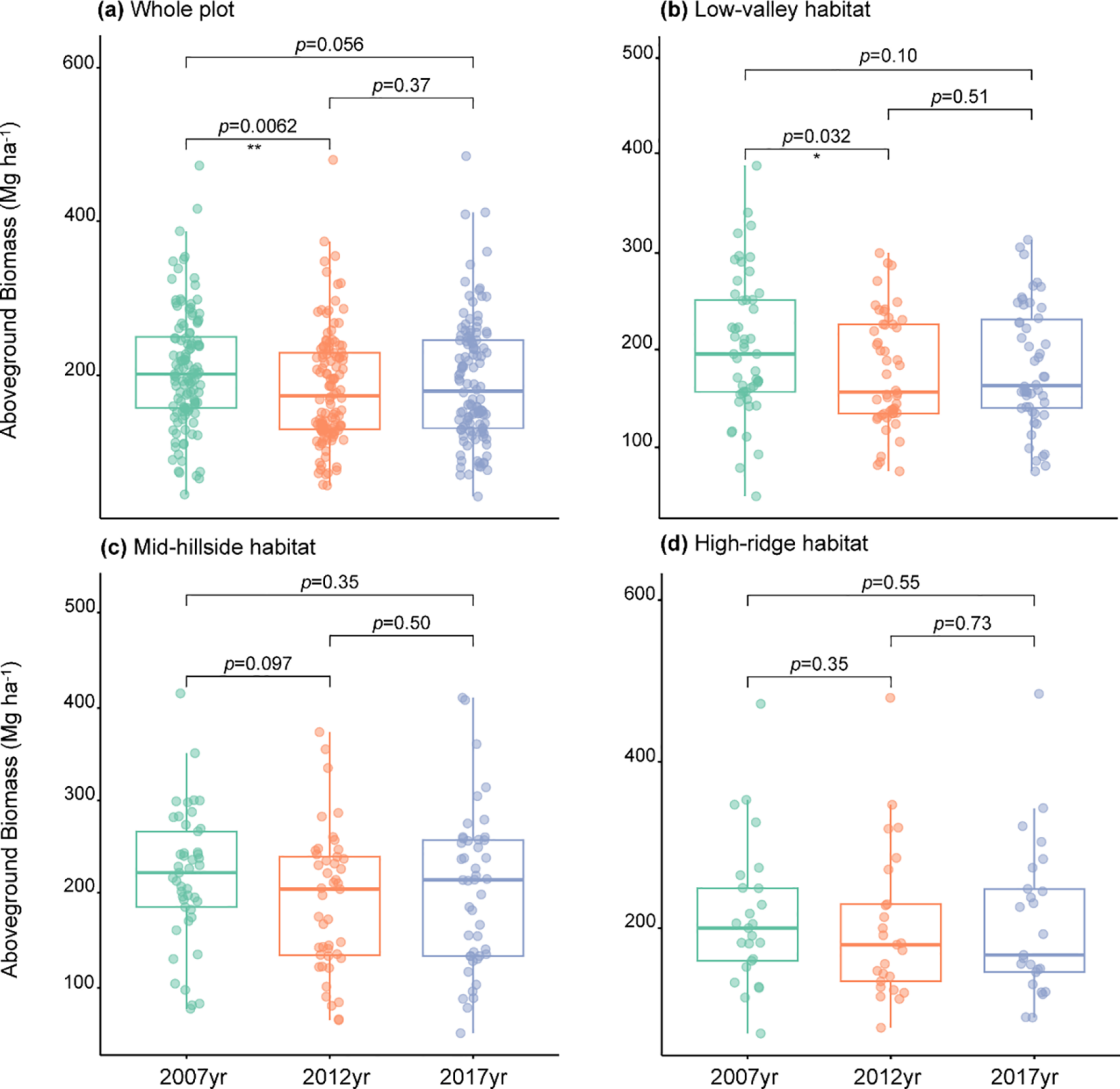
The aboveground biomass changes of the whole plot **(A)**, Low-valley habitat **(B)**, Mid-hillside habitat **(C)** and High-ridge habitat **(D)** from 2007 to 2017. *p< 0.05, **p< 0.01, ***p< 0.001.

By estimating the AGB in Gutianshan 5-ha plot, we found that the AGB at community level showed a significant decrease followed by a nonsignificant increase from 2007 to 2017 (**Table 2**, **Figure 1A**), which was mainly driven by the biomass change in low-valley habitat (H1) (**Table 2**, **Figure 1B**). The AGB in the mid-hillside (H2) and high-ridge habitats (H3) from 2007 to 2017 also showed a decreasing and then increasing change, but variations at each stage were not significant (**Table 2**, **Figures 1C, D**).

**Table 2:** The aboveground biomass of the whole plot and different habitat types from 2007 to 2017.

Year	Whole plot	Low-valley habitat	Mid-hillside habitat	High-ridge habitat
Aboveground biomass (± sd) (Mg ha^-1^)				
2007	212.45 (± 75.37)	204.84 (± 71.38)	219.00 (± 71.96)	215.87 (± 89.77)
2012	188.27 (± 73.20)	178.33 (± 57.62)	191.86 (± 76.26)	201.69 (± 93.33)
2017	195.89 (± 78.57)	183.10 (± 62.04)	204.01 (± 85.52)	206.87 (± 93.56)

### 3.2 The changes of drivers influencing aboveground biomass dynamics over time

**Figure 2:**
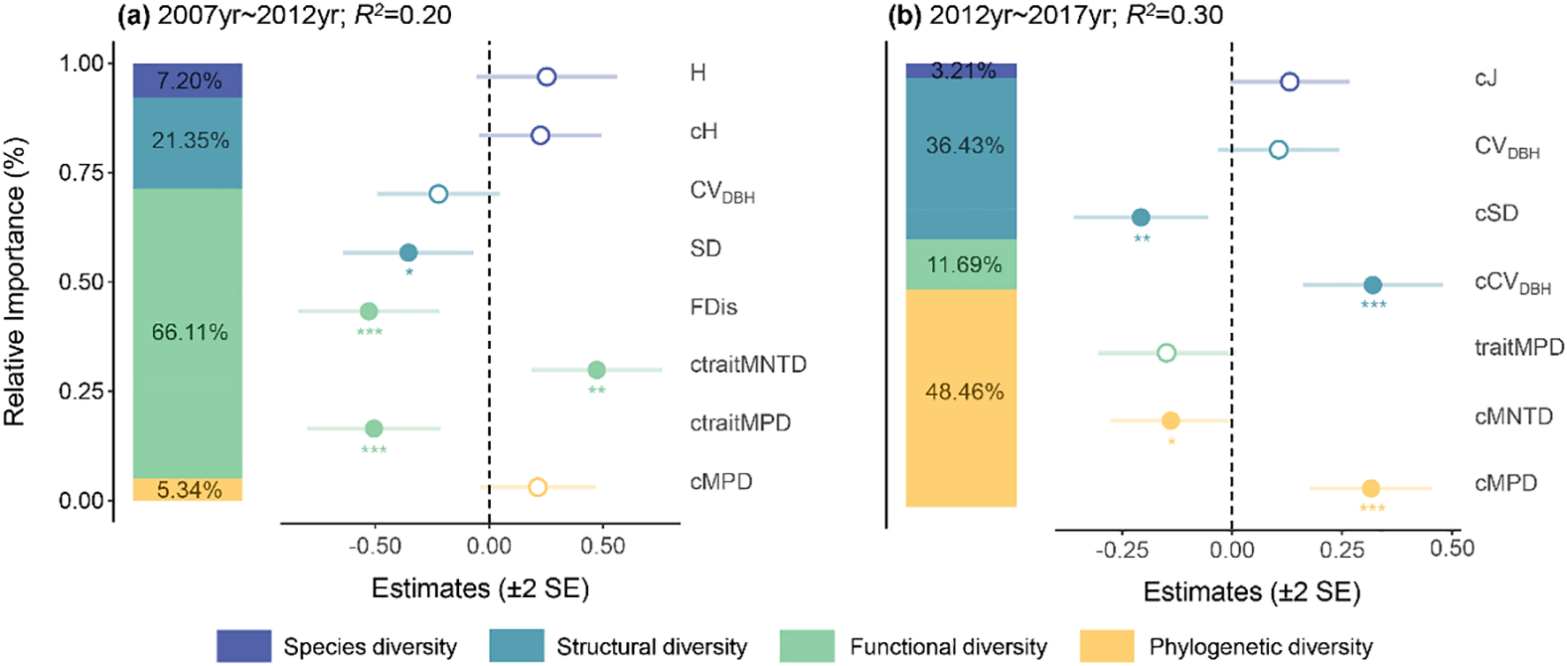
The main impact factors of aboveground biomass dynamics from 2007 to 2012 **(A)** and 2012 to 2017 **(B)**. Solid and open circles indicate significant or nonsignificant community diversity effects at p< 0.05, respectively. H, Shannon-Wiener index; CV_DBH_, coefficient of variation in DBH; SD, stand density; FDis, functional dispersion; MPD, mean pairwise distance; MNTD, mean nearest taxon distance; traitMPD and traitMNTD, trait mean pairwise distance and trait mean nearest taxon distance; J, Pielou evenness index. c indicates the change values of each variable. *p< 0.05, **p< 0.01, ***p< 0.001.

The main impact factors on ΔAGB were functional diversity (66.11% of relative importance) and structural diversity (21.35% of relative importance) during 2007-2012 period (**Figure 2A**). Specifically, ΔAGB was significantly negatively correlated with SD, FDis and ctraitMPD, but significantly positively correlated with ctraitMNTD. However, during 2012-2017 period, ΔAGB was mainly affected by phylogenetic diversity (48.46% of relative importance) and structural diversity (36.43% of relative importance) (**Figure 2B**). It showed a significant positive relationship with cMPD and cCV_DBH_, but a significant negative relationship with cSD and cMNTD.

### 3.3 The changes of drivers influencing aboveground biomass dynamics across habitats

**Figure 3:**
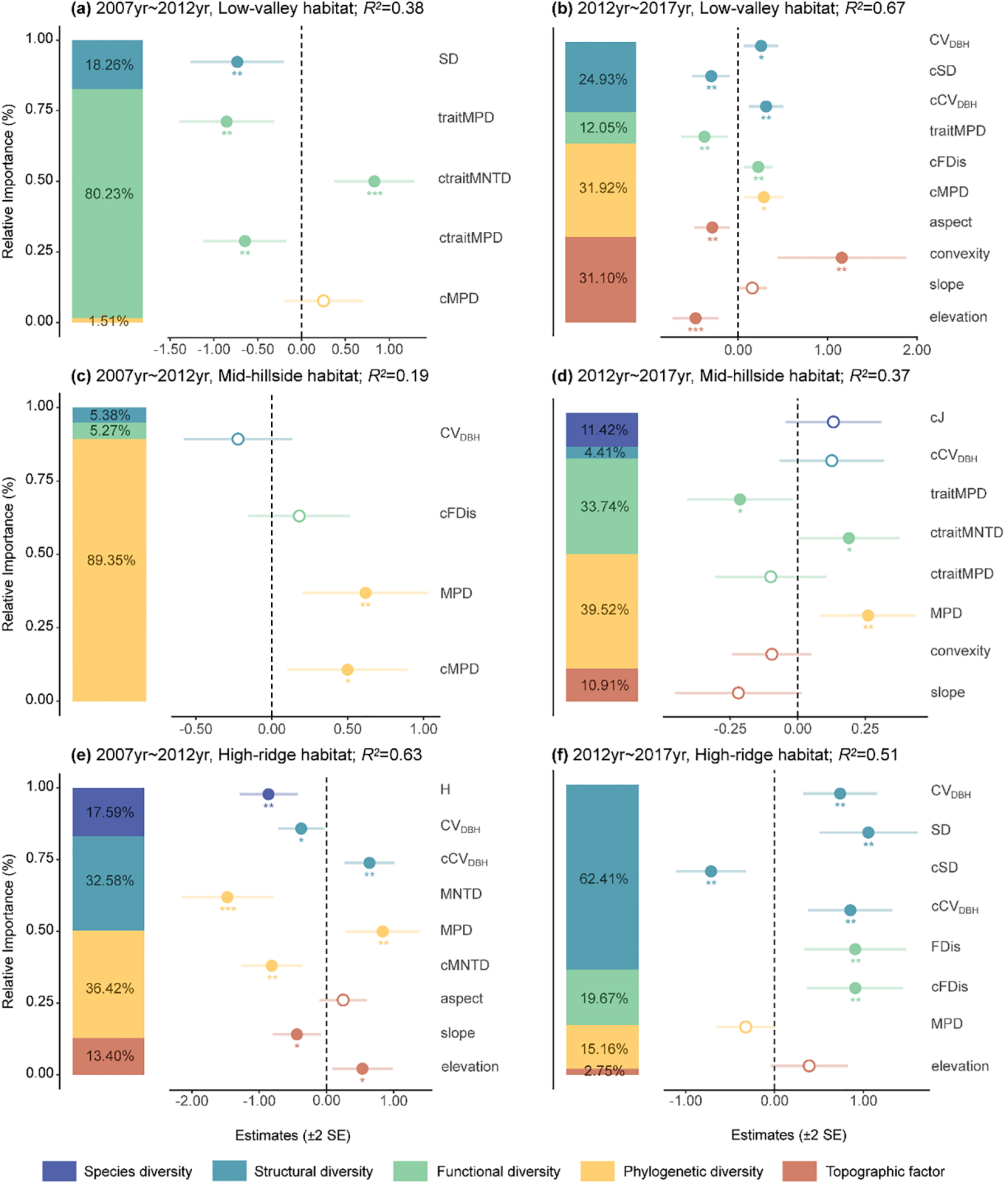
The main impact factors of aboveground biomass dynamics in Low-valley habitat **(A, B)**, Mid-hillside habitat **(C, D)**, and High-ridge habitat **(E, F)** from 2007 to 2017. Solid and open circles indicate significant or nonsignificant community diversity effects at p< 0.05, respectively. The meanings of abbreviations are the same as in **Figure 2**. *p< 0.05, **p< 0.01, ***p< 0.001.

The main impact factors on ΔAGB in low-valley habitat were similar to the whole plot (**Figures 3A, B**). Moreover, we also found that it was significantly negatively correlated with aspect and elevation, but significantly positively correlated with convexity (**Figure 3B**). ΔAGB in mid-hillside habitat was significantly positively affected by phylogenetic diversity (**Figures 3C, D**). Besides, ΔAGB was significantly negatively associated with traitMPD but significantly positively associated with ctraitMNTD (**Figure 3D**). In high-ridge habitat, all dimensions of biodiversity as well as topographic factors showed significant effects on ΔAGB (**Figures 3E, F**). Specifically, ΔAGB was significantly negatively influenced by H, MNTD, cMNTD, cSD and slope, but was significantly positively influenced by cFDis, FDis, cCV_DBH_, MPD, SD and elevation. Whereas CV_DBH_ had a variable relationship with ΔAGB.

## 4 Discussion

### 4.1 Changes of aboveground biomass over the decade

The AGB at community level in Gutianshan showed a significant decrease during the 2007-2012 period. The large-scale ice storm occurred in this region in 2008 killed many trees especially the larger-diameter trees with higher biomass (Jin et al., 2015), which might cause a significant decrease in AGB of the plot (Zhang et al., 2012). During the 2012-2017 period, the AGB increased but did not recover to the initial level, which was probably due to the stable forest type here. Most of the forest here is in the middle and late successional stages, with well-developed, typical, and stable vegetation, belonging to a mature subtropical evergreen broad-leaved forest (Legendre et al., 2009). However, the more stable a forest is before a disaster, the slower it recovers afterwards (Sun et al., 2012). Furthermore, the restoration of some forest ecosystem functions, such as the AGB and carbon sequestration, could span decades or potentially even longer periods (Amiro et al., 2010).

After examining the AGB across the three habitats within the plot, our results revealed that the trend of AGB variation at the community level was primarily driven by changes in low-valley habitat. In contrast, the AGB in mid-hillside and high-ridge habitats showed nonsignificant variation, indicating that the damage to trees in low-altitude valley was more severe than in mid- and high-altitude regions (Man et al., 2011). This was not aligned with the established impact of natural disasters on forest vegetation (Zhang et al., 2012; Tanner et al., 2014). The discrepancy may be due to the fact that the elevation differences within this plot are not substantial enough to reflect the influence of altitude. Additionally, the impact of elevation on the severity of damage to forest vegetation after disasters can be shaped by the distinctive characteristics of the local environment (Man et al., 2011).

### 4.2 Changes of drivers and effects on aboveground biomass dynamics across temporal scales

Changes in community performance may be attributed to plant ecological strategies, which impact the efficacy and interplay of species, thereby affecting the ecological processes and functions of ecosystem (Huang et al., 2018; Zheng et al., 2024). Our study showed that the factors influencing ΔAGB varied across time scales. During the 2007-2012 period, the main influencing factors on ΔAGB were functional diversity and structural diversity. It is generally believed that both functional diversity and structural diversity are beneficial for increasing ecosystem biomass accumulation or productivity (Li et al., 2019; Lian et al., 2022) as both can promote the resource use efficiency of the community (Zhu et al., 2021; Chen et al., 2023). However, in our study, functional diversity and structural diversity were mainly negatively correlated with ΔAGB. This is likely because of the negative relationship between plant diversity and resource availability in natural ecosystems due to resource constraints and interspecific competition (Fraser et al., 2015). The communities in the study plots are mostly in the middle and late successional stages, where resources are relatively limited (Lasky et al., 2014). In addition, the increase of plant diversity and individuals at this stage tended to intensify interspecific competition (**Table 1**), which further reduced the available resources for species and ultimately resulted in a decrease of community productivity (Wu et al., 2018). As a result, a negative BEF relationship occurred during this period.

In the subsequent period of 2012-2017, we found that the main impact factors on ΔAGB shifted to phylogenetic diversity and structural diversity. This is in line with previous studies suggesting that BEF relationships in forests could change over time (Lasky et al., 2014; Gottschall et al., 2022). The shift could be a consequence of the formation of canopy gaps in this forest (Man et al., 2011), which could increase the light availability of understory vegetation (Zhu et al., 2014; Song et al., 2018). This increase could promote the recruitment of early-successional species that struggle to reproduce under low light conditions, as well as the regeneration of late-successional species (Song et al., 2018). As a result, phylogenetic diversity might have encapsulated certain inherent functional characteristics that were not directly assessed during this period, including traits related to roots or herbivores (Cadotte et al., 2009; Liu et al., 2015). The ΔAGB was mainly positively correlated with phylogenetic diversity and structural diversity during the 2012-2017 period. This could be attributed to the balance achieved between the high productivity but high mortality rates of early-successional species (acquisition strategy), and the lower productivity but also low mortality rates of late-successional species (conservative strategy), resulting in an increase in community productivity (Lasky et al., 2014; Zhang et al., 2023). However, we also found that ΔAGB was significantly negatively correlated with cSD and cMNTD. The variability observed in the correlations between various biodiversity indices and ΔAGB could be due to the varying capacities of each metric to capture the intensity of interactions within the forest ecosystems being studied, rather than an inherent ecological process (Yuan et al., 2018). Our results underscore the important role of multidimensional biodiversity and community context in elucidating the dynamic BEF relationships across temporal scales.

### 4.3 Changes of drivers and effects on aboveground biomass dynamics across habitat types

The spatial heterogeneity in resource supply rates can directly influence the biomass of producers, or indirectly impact producer biomass by limiting the variety of species that can coexist within an ecosystem (Cardinale et al., 2009; Ferry et al., 2010). Our results revealed that both the drivers and effects on ΔAGB significantly varied across different habitat types. The factors influencing ΔAGB in low-valley habitat were extremely similar to those of the whole plot, possibly because this habitat has the highest number of plots (50), closely resembling the resource supply and utilization patterns of the whole plot. The result does not align with the discoveries from the low-altitude area in Dinghai, Zhejiang Province, where a nonsignificant relationship was found between biodiversity and biomass or productivity (Wu et al., 2018). This inconsistency may be a result of different dimensions of biodiversity, or the complex mediating role of environmental factors in the BEF relationship (Zhu et al., 2021). Additionally, we found that ΔAGB was significantly negatively correlated with aspect and elevation, but significantly positively correlated with convexity in this habitat. The low-valley habitat is defined by its significant topographical heterogeneity, featuring prominent rocks and small streams, making it particularly prone to regular disturbances like tree falls and seasonal stream flooding (Xu et al., 2015). Therefore, topographic factors had a significant effect on ΔAGB in this period.

In mid-hillside habitat, ΔAGB mainly showed a significant positive relationship with phylogenetic diversity. Liu et al. (2022) also found that phylogenetic diversity reaches its maximum in the mid-elevation region. It may be because the mid-hillside habitat serves as a transitional area between the low-valley and high-ridge habitats, where the favorable supply of light and water resources allows for the growth of most species in this environment (Legendre et al., 2009; Coomes et al., 2014). In addition, there were many broken and uprooted large trees found in slopes perhaps due to steep hillsides and shallow soil (Ferry et al., 2010; Xu et al., 2015), which could increase the openness of the canopy gaps, resulting in rapid regeneration of understory species (Song et al., 2018). Such an environment might offer opportunities for species with greater phylogenetic distance and different life history strategies to survive. In summary, it can be concluded that such ecological environment and resource supply enhance the positive effect of phylogenetic diversity on ΔAGB.

In high-ridge habitat, we found that ΔAGB was significantly influenced by all dimensions of biodiversity and topographical factors, which is consistent with the findings of most studies (Lasky et al., 2014; Yuan et al., 2018; Tiwari et al., 2023). The relationships among species in high-ridge habitat (high-altitude area) tend to be more intimate (Liu et al., 2022). The canopy gaps might increase the opportunities for species less associated with the vegetation in high-ridge habitat to recolonize from neighboring areas (Roxburgh et al., 2004). Given that the habitat is less disturbed (Man et al., 2011), the diversity of communities could reach the maximum and the coexistence of species will be promoted according to the Intermediate Disturbance Hypothesis (IDH) (Roxburgh et al., 2004). Moreover, the competition for resources especially light is minimal due to the lower stand density within high-ridge habitat (Ullah et al., 2021; Su et al., 2023), providing favorable conditions for the growth of recolonizing species. Overall, the increase in diversity had enhanced their influence on ΔAGB (**Table 1**). Synthesizing the results garnered from the various habitat types examined, our findings highlight that habitat heterogeneity constitutes a pivotal driver influencing the BEF relationship, providing a plausible perspective for investigating the spatial variations in BEF relationships.

## 5 Conclusions

In the present study, our comprehensive analysis elucidates the intricate interplay between multidimensional diversity and ΔAGB within natural forest ecosystems. We have demonstrated that both abiotic factors, such as topography, and biotic factors including functional diversity, phylogenetic diversity and structural diversity exert a significant influence on the ΔAGB over time and across various habitat types. Our decade-long analysis revealed a notable decline followed by an increase in community-level AGB, primarily within low-valley habitat, with no significant alterations observed in mid-hillside and high-ridge habitats. The determinants of ΔAGB exhibited substantial temporal shifts; functional and structural diversity were pivotal during the earlier period, while phylogenetic and structural diversity became increasingly influential in the subsequent period. Moreover, the drivers and effects on ΔAGB significantly varied across different habitat types. Our findings underscore the necessity of considering the spatiotemporal variability of both abiotic and biotic factors when assessing ecosystem function. This study not only addresses a critical gap in our understanding of BEF relationships but also provides valuable insights for conservation and management strategies aimed at preserving the health and resilience of forest ecosystems.

## Data Availability

The original contributions presented in the study are included in the article/supplementary material. Further inquiries can be directed to the corresponding author.
